# Process evaluation and assessment of use of a large scale water filter and cookstove program in Rwanda

**DOI:** 10.1186/s12889-016-3237-0

**Published:** 2016-07-16

**Authors:** Christina K. Barstow, Corey L. Nagel, Thomas F. Clasen, Evan A. Thomas

**Affiliations:** Civil, Environmental and Architectural Engineering, University of Colorado, Boulder, CO USA; School of Public Health, Oregon Health and Science University, Portland State University, Portland, OR USA; Department of Environmental Health, Rollins School of Public Health, Emory University, Atlanta, GA USA; Mechanical and Materials Engineering, Portland State University, Portland, OR USA

## Abstract

**Background:**

In an effort to reduce the disease burden in rural Rwanda, decrease poverty associated with expenditures for fuel, and minimize the environmental impact on forests and greenhouse gases from inefficient combustion of biomass, the Rwanda Ministry of Health (MOH) partnered with DelAgua Health (DelAgua), a private social enterprise, to distribute and promote the use of improved cookstoves and advanced water filters to the poorest quarter of households (*Ubudehe* 1 and 2) nationally, beginning in Western Province under a program branded *Tubeho Neza (*“Live Well”). The project is privately financed and earns revenue from carbon credits under the United Nations Clean Development Mechanism.

**Methods:**

During a 3-month period in late 2014, over 470,000 people living in over 101,000 households were provided free water filters and cookstoves. Following the distribution, community health workers visited nearly 98 % of households to perform household level education and training activities. Over 87 % of households were visited again within 6 months with a basic survey conducted. Detailed adoption surveys were conducted among a sample of households, 1000 in the first round, 187 in the second.

**Results:**

Approximately a year after distribution, reported water filter use was above 90 % (+/−4 % CI) and water present in filter was observed in over 76 % (+/−6 % CI) of households, while the reported primary stove was nearly 90 % (+/−4.4 % CI) and of households cooking at the time of the visit, over 83 % (+/−5.3 % CI) were on the improved stove. There was no observed association between household size and stove stacking behavior.

**Conclusions:**

This program suggests that free distribution is not a determinant of low adoption. It is plausible that continued engagement in households, enabled by Ministry of Health support and carbon financed revenue, contributed to high adoption rates. Overall, the program was able to demonstrate a privately financed, public health intervention can achieve high levels of initial adoption and usage of household level water filtration and improved cookstoves at a large scale.

**Electronic supplementary material:**

The online version of this article (doi:10.1186/s12889-016-3237-0) contains supplementary material, which is available to authorized users.

## Introduction

Contaminated air and drinking water at the household level are significant contributors to morbidity and mortality among rural populations in low-income countries. Household air pollution (HAP) contributes to acute lower respiratory infection (ALRI), the leading cause of death in children under 5 [1]. Among adults, HAP is a risk factor for ischaemic heart disease, stroke, hypertension, chronic obstructive pulmonary disease, lung cancer, trachea, bronchus, cerebrovascular disease and cataracts [2–4]. HAP from indoor cooking with solid fuels (coal, wood, charcoal, dung and agricultural residues) is responsible for 18 % of global burden of disease in 2012; indoor cooking is also linked to a half million deaths annually from outdoor air pollution [5]. Inadequate and unsafe drinking water is the leading cause of diarrheal disease, which alone accounts for more than 1.4 million deaths annually. Collectively, pneumonia and diarrhea are responsible for an estimated 6.9 million deaths annually [1].

These environmental hazards are further aggravated among impoverished rural inhabitants of sub-Saharan Africa, the vast majority of whom cook with biomass fuels on traditional stoves and rely on unsafe water supplies. In Rwanda, where more than half the population is living below the poverty line and more than a third in extreme poverty, 99.0 % of rural householders cook with biomass, mainly on open three-stone fires, and only 2.2 % have water on their premises [6]. In Rwanda, leading causes of death in children under five include ALRI (16 %) and diarrhea (9 %) [7].

Household environmental health interventions like water filters and improved cookstoves, combined with on-going comprehensive household engagement, may help address these health issues [3, 8]. However, the up-front cost of household filters and cookstoves, together with the need to establish supply chains for consumables, has limited the extent to which they have been scaled up among vulnerable populations, particularly in rural settings. Published studies that have evaluated household water treatment and improved cookstove interventions often describe efforts implemented on the thousands or hundreds of households scale. There are few published journal articles known to the authors that rigorously describe programs on the scale of more than 100,000 households.

## Background

In an effort to reduce the disease burden in rural Rwanda, decrease poverty associated with expenditures for fuel, and minimize the environmental impact from inefficient combustion of biomass, the Rwanda Ministry of Health (MOH) partnered with DelAgua Health (DelAgua), a private social enterprise, to distribute and promote the use of improved cookstoves and advanced water filters to the poorest quarter of households (*Ubudehe* 1 and 2) nationally, beginning in Western Province. The project is privately financed and earns revenue from carbon credits under the United Nations Clean Development Mechanism (CDM), a program authorized by the Kyoto Protocol that provides market-priced credits to the implementer based on a formula that includes population coverage and use [9].

DelAgua and MOH first undertook a pilot intervention (Phase 1) to all 1943 households in 15 rural villages working with recruited Community Health Workers (CHWs) [10]. The London School of Hygiene and Tropical Medicine then undertook a 5-month cluster randomized trial among 566 households in three pilot villages to assess coverage and use, the impact of the water filter on fecal indicator bacteria in household drinking water and the impact of the stove on fine particulate matter (PM_2.5_) and carbon monoxide (CO) in reported cooking areas [11]. While reported filter use was high (89.2 %), 25 % reported drinking from other sources at least once during 5 follow-up visits; filter-mounted sensors also showed self-reports to exaggerate use [12]. Overall, the intervention was associated with a 97.5 % reduction in mean faecal indicator bacteria at the point of consumption (Williams means 0.5 vs. 20.2 TTC/100 mL, *p* < 0.001). Two-thirds (66.7 %) of intervention households identified the intervention stove as their main cooking stove, but only 23.3 % of intervention households reported that their main cooking area was outdoors. Overall, the stoves were associated with a 48 % reduction of 24-h PM_2.5_ concentrations in the cooking area (0.485 mg/m^3^ and 0.267 mg/m^3^, *p* = 0.005). The reduction was 37 % for those cooking indoors (*p* = 0.08) and 73 % for those cooking outdoors (*p* < 0.001) [11]. Following the pilot RCT, 9 of the non-RCT pilot villages were matched with control villages and followed for an additional 12 months to assess longer-term intervention uptake and to test methods for assessing exposure and health outcomes for a larger scale health impact evaluation. The results of the matched cohort study are still being analyzed.

The Phase 1 effort yielded several lessons integrated into the large-scale Phase 2 program. These included design improvements to both the stoves and filters in collaboration with the manufacturers, improved criteria for CHW selection, interactive materials for household education, and targeted curriculum for exclusive and consistent use of both the stoves and filters.

Based on the results from the pilot study, DelAgua and the MOH elected to proceed with the roll out of the intervention throughout the Western Province of Rwanda (Phase 2). For logistical and research purposes, it was agreed that 70 of the 96 sectors (groups of villages that also correspond with catchment areas for primary care clinics) would be covered in the initial round of implementation (September through December 2014); 24 sectors that would be covered later serve as the control group in a sector-level RCT to assess the impact of the intervention on health outcomes (ClinicalTrials.gov, NCT02239250). Two sectors were excluded after a field study determined greater than 50 % of *Ubudehe* 1 and 2 households in these areas were primary charcoal users for which the stoves were less suitable.

## Methods

### Program description

**Fig. 1:**
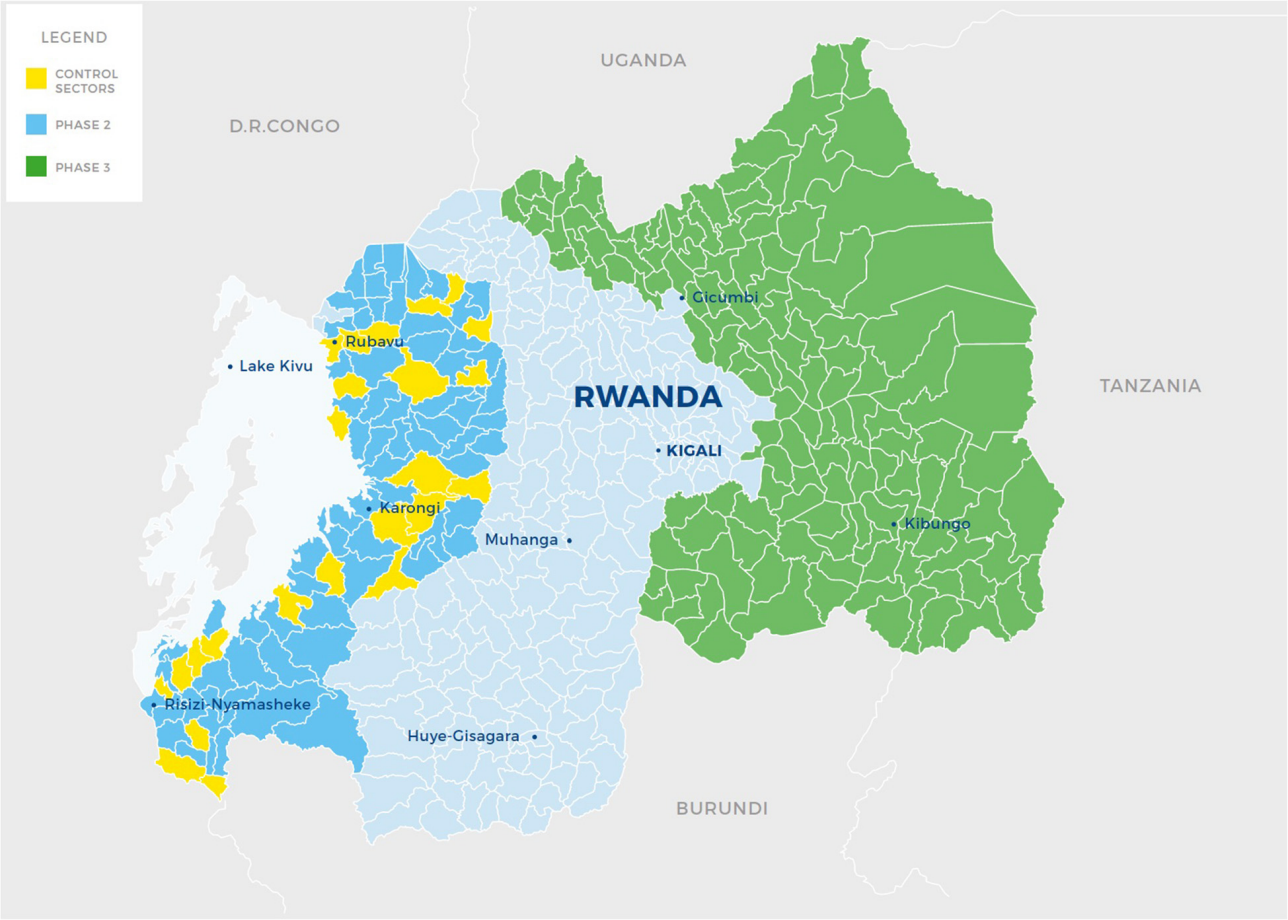
Rwanda with Sector administrative boundaries. Phase 2 Tubeho Neza distributions occurred in the dark blue sectors in the Western Province. Yellow regions identify control sectors. Planned Phase 3 activities in 2016 are highlighted in green predominately in the Eastern Province. Figure copyrighted by, and reprinted with permission from, DelAgua Health Limited

The program is branded *Tubeho Neza* which means to “Live Well” in Kinyarwanda. *Tubeho Neza* includes the distribution of the Vestergaard Frandsen LifeStraw Family 2.0 household gravity-fed water filter and the EcoZoom Dura high efficiency wood cookstove, and associated community and household education and behavior change messaging. The intervention technologies have been described elsewhere [11]. Recipients of the technologies included all households classified as *Ubudehe* 1 and 2 (the government-recognized poorest 25 % of the country) in 70 of 96 sectors in the Western Province (Fig. 1). Technologies were also distributed to local leaders including all CHWs, village chiefs and cell level (2–16 villages) officials in intervention areas. All households originally enrolled in the Phase I effort were integrated into the *Tubeho Neza* program and received upgraded filters and cookstove servicing. Figure 1 shows expansion plans for Phase 3, in 2016.

Leveraging several behavior change methodologies as described in the pilot study [10], the program provides informational and education contact to the beneficiary at multiple key points to facilitate the adoption and sustained use of the water filter and improved cookstove. Activities include a social marketing campaign before and during distributions, community level product delivery facilitated by local leaders, and household level education performed by CHWs immediately after the household receives the products with ongoing visits at regular intervals.

### CHW selection and training program

Rwanda’s extensive CHW network includes three CHWs for each village with nearly 11,000 CHWs in the Western Province of Rwanda. CHWs were selected based on criteria including Kinyarwanda literacy (the national language), timeliness, responsiveness, smartphone competence and program knowledge.

CHW trainings were conducted in each of the seven Western Province Districts with an average of ten CHW Sector teams and 124 CHW participants per training. Each training included 2.5 days of lessons with topics related to the use and maintenance of the technologies, smart phone and application based data collection, basics of survey enumeration and communication and engagement with household members, with emphasis on learner-participatory, interactive techniques. Lessons were designed with as much hands-on and practice based learning as possible, partly to impart knowledge to CHWs in the most effective manner, but also to model the engagement method. Specifically, use of a smart phone was known to be a challenging skill for many CHWs, and thus over 40 % of the training revolved around learning smart phone based skills. Additional importance was placed on non-exclusive engagement with both genders and across all age ranges.

### Social marketing

To create awareness around the program and provide initial knowledge for the products, radio advertisements and sensitization meetings were conducted before households received their water filters and improved cookstoves. Advertisements were run on three different regional radio stations with combined reach across the majority of Western Province. Two different radio scripts were aired, one before the start of the campaign and the second during distributions. The first advertisement focused on creating awareness around the *Tubeho Neza* campaign itself and the adverse health and environmental effects of indoor air pollution and contaminated water. The second advertisement then focused on the benefits of the products and the positive effects of product use on a household.

Sensitization meetings were conducted by the South African marketing agency, EXP, anywhere from 1 day to 2 weeks before the households in the community were to attend the distribution meeting to collect their products. The focus of these meetings was to introduce the larger community to the program, while providing initial exposure to the water filter and improved cookstove before households received them. Demonstrations of the products’ use were conducted emphasizing potential benefits, with the aim of generating excitement and initial user knowledge. Finally, local authorities took the opportunity to communicate the date and location of the upcoming distribution meeting, as well as reach out to the specific targeted households, in order to maximize turnout for the distribution meeting.

### Distributions

Prior to distributions, 360 unique distribution points were identified, including government offices, schools, churches and health facilities. In addition, an extensive process of updating the beneficiary list was completed before distributions. The list identifying the *Ubudehe* category for each household was completed in 2012, 2 years before the program. These lists were distributed to village chiefs who were asked to update them based on the current residents of his/her village. After all storage and distribution points and the schedule were established, the Rwanda National Police were responsible for transporting products from the capital to the 360 established locations.

Each distribution was facilitated by local officials who gave opening remarks regarding the program. CHWs then performed a skit that portrayed a family before and after receiving the water filter and cookstove. The skit ends with the singing and dancing of the *Tubeho Neza* song. After the skit, households were asked to queue in order to receive the products. Discrepancies or disagreements on distribution lists were arbitrated by village chiefs or local CHW leaders. A separate smartphone-based distribution form was collected for each household which included household identification information, photos and signature of recipients, and barcode scanning of the water filter and cookstove. Households were then instructed to bring their products home and wait to be visited by a CHW.

Distributions occurred throughout the Western Province, starting with four distributions in the first week and reaching 59 distributions at the peak of the campaign. On average 31 distributions were conducted per week during the 13 weeks of the campaign. Distributions occurred at the cell level, which on average consists of seven villages. The size of a distribution varied from 25 to 753 households with an average of 256 households per distribution. Given the varying size of a particular cell, distributions took anywhere from several hours to 2 days.

### Initial household visit

Following distribution activities, CHWs convened with their DelAgua supervisor to divide up household clusters and visiting routes, devising a strategy for completing all household visits, with input and sometimes accompaniment from authorities most familiar with the particular areas. Rwanda’s challenging terrain often meant CHWs had to travel distances of several kilometers to reach beneficiary households. Household visits were performed for a total of 98 days with an average of 1037 household visits performed each day. On average 79 CHWs were performing household visits 6 days a week. At the peak of the program 309 CHWs performed 2274 household visits in a single day. Visits were tracked through a smart phone based form, which could be cross referenced with other parameters in the distribution forms to determine any households who received products at distribution but had not yet been visited by a CHW. As with the distribution forms, additional analysis was performed to identify duplicate household visits or other possible data entry errors.

Household visits included two components; a brief baseline survey and an extensive education and training session. The survey included baseline fuel, stove, cooking location, water source and any water treatment methods currently used by the household taking approximately 15 min to administer. Additionally general household identifying information was collected (names, phone numbers, identification numbers, GPS coordinates) and product barcodes of the newly received filter and cookstove were scanned to track products to specific household locations.

**Fig. 2:**
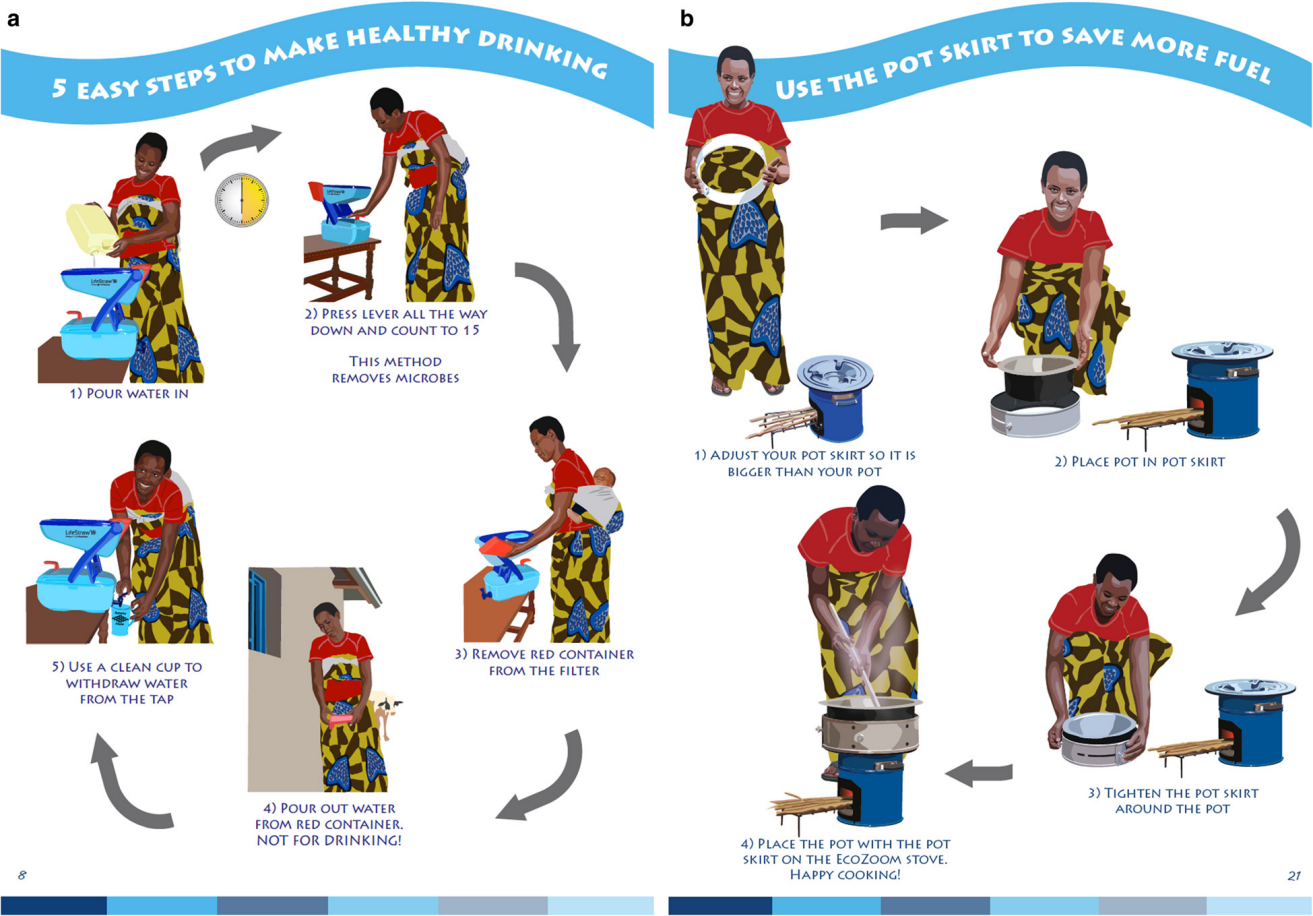
Example pages from educational flipbook used by CHWs during household education visits. Figure copyrighted by, and reprinted with permission from, DelAgua Health Limited

Household education included use of interactive teaching tools, primarily an illustration based flipbook and a poster, customized to the household’s size and daily routines, which was hung in each household. The design of the flipbook included colorful graphic images illustrated from photographs (example pages shown in Fig. 2). Images were piloted with several families to develop appealing and culturally appropriate images. Each page of the flipbook included a specific message to be communicated to the family by the CHW. Instructional pages included a step-by-step process to perform usage and maintenance tasks, while prompting the CHW to physically perform the tasks and have members of the family demonstrate usage. Households had been advised during the distribution meeting to fill the water filter in preparation for the CHW visit, as the initial filling of the backwashing chamber might in some cases exceed household visit time, so that this maintenance feature could be demonstrated with full functionality. The poster included several activities personalized for each family such as circling the number of times to fill a filter in order to provide the entire recommended water consumption amount to all members of a family per day based on its size.

Key messages included:

- Family Oriented - Both the flipbook and poster emphasized ownership of the products by all members, aspiring to be a healthy and happy *Tubeho Neza* family. CHWs were encouraged to engage all available members of a household in the visit.
- Health, Environment and Livelihood Consequences and Benefits – Common diseases and health effects from contaminated drinking water and indoor air pollution were highlighted, as well as possible environmental effects from deforestation. Many of these consequences were then discussed in relation to benefits from using the technologies including financial savings, time savings and cleanliness.
- Comprehensive Filter Description – Phase I households expressed interest in understanding exactly how the filter worked as it was seen as intimidating which made some households hesitant to use and adopt the product. A pictorial description of membrane filtration and the cleaning process was added which helped households understand the importance of backwashing.
- Hydration – In response to skepticism from Phase I households over the program’s messaging of the importance of consumption of two liters of water per person per day, messaging was developed to promote hydration through explanation of its health benefits, including reinforcement of the biological importance of water for all ages, young and old.
- Exclusive Use of Filtered Water – Targeted messaging was developed to encourage families to bring filtered water with them to school, work or leisure activities. Families were also asked to designate clean containers as *Tubeho Neza* containers to be used only for safe water storage. A hatch mark was drawn on the containers to distinguish these from others, and households were trained to clean such containers once a week.
- Wood Storage – The difficulty in using the EcoZoom stove with wet or damp wood was indicated by many Phase I households. Households were asked to designate a specific area where fuelwood could be stored so that it could be dry for future use.
- Stove Stacking Behavior – To combat stove stacking (use of traditional stove alongside improved stove), examples of reduced cooking times and fuel consumption from using the improved cookstove were emphasized in the flipbook as well as negative messaging around the use of the traditional three stone fires being harmful and wasteful.
- Cooking Location – To provide additional health benefits related to the use of the improved cookstove, households were instructed to cook outside. However, this was difficult for many households with the large amount of precipitation in much of the Western Province. Therefore, messaging highlighted the portability of the stove, to show it could be moved to a doorway or other household location both well-ventilated and covered.

When CHWs finished the education lesson, beneficiaries were asked to countersign an agreement between their household and a local official acknowledging that the products are for the benefit of the family and are not for sale. A record of this agreement was kept by photographing it using the smart phone. Additionally a shortcode for a repair and replacement hotline was displayed on the poster, which families can contact in case of any problems with the products.

### Follow up household visits

Following the 2014 distribution, a follow up campaign was implemented which consisted of household visits to all households who originally received products. The follow up visits were conducted in the Spring of 2015 between 6 weeks and 6 months after households received products. All CHWs were deployed within a 5-week time period. On average CHWs performed 1176 household visits per day with a peak of 3557 households visited by 604 CHWs in a single day.

A follow up household visit included a brief survey to assess several adoption and programmatic metrics, repair and replacement of broken products, cleaning of the filter’s bottom safe storage water container and an education and training lesson.

The CHW follow up survey questions were focused on current water treatment and cooking practices, primarily assessing initial adoption and continued use of the filter and cookstove. Questions included asking households to report their current household behaviors but additional observational measures were included such as the presence of water in a filter or visible cooking practices occurring during the visit to provide more objective data points. The survey was of similar length to the initial household survey and could be administered in approximately 10 min.

The household education included emphasis of critical messaging as described previously through similar picture based images presented through a new education material, a yearly calendar, with messaging resembling that used in the original flipbook and poster. Prominence on the calendar was accorded to specific messaging components based on relative priorities of re-visiting, taken from an analysis of the previously mentioned assessment surveys conducted in the quality control activities of the initial household visit campaign. Households were encouraged to use the calendar for their daily lives, as well as events related to the technologies, such as weekly or monthly cleaning tasks. Household members present at the time of visit were again asked to demonstrate use of the products, and CHWs ensured they were able to perform all necessary tasks. Additionally, a *Tubeho Neza* designated safe water sticker, with an illustration of the model *Tubeho Neza* family, was added to safe storage devices previously designated with the *Tubeho Neza* hatch symbol. This was intended, not only to reinforce sanitation behaviors associated with the filter, but also to encourage pride in households’ self-identification with the *Tubeho Neza* program when using the safe storage devices out in the community. Finally, all households deemed by the CHW to be correctly using and maintaining the technologies were given a plastic *Tubeho Neza* bracelet as a token of further identification with the program.

To track the follow up campaign, supervisors used a comprehensive smart phone reporting system. Any household that could not be found was reported for as missing, moved or otherwise unavailable, by supervisors while any unaccounted for product was reported as missing, moved or otherwise. Any product that could not be repaired by CHW’s at the time of the visit was reported by the CHW as “in need of repair” in a section in the survey. DelAgua supervisors provided CHW teams with certain filter replacement parts, including taps, backwashing tubes, backwashing container and pre-filters, to be used in CHW-repairs, which were also tracked through the Follow Up Survey. Additionally, in households found to have sold or attempted to sell one or both of the products, or in households found to have mistakenly received product, the product was repossessed by a Supervisor, and returned to a local storage facility.

To combat potential algae growth in the bottom container of the filter, as seen in some Phase I households, a mandatory cleaning of each of the filters was performed by CHWs. Households were not instructed to clean the filters themselves, as this might introduce contamination.

### On-going promotion activities

Behavior change and reinforcement activities are ongoing throughout the intervention area. DelAgua staff reside full time in each of the seven Districts of Western Province to manage these activities. Ongoing behavior change activities include:

- CHW Cooperative Meetings – Staff provide additional educational messaging, receive updates on adoption within households and facilitate incorporation of the *Tubeho Neza* program into other health programs.
- Community Meetings – Staff carry out informational sessions which address specific educational goals at common community meetings such as the community service day (*Umuganda*), market days and other official meetings, as well as to provide repair and replacement services.
- Field and Household Visits – Staff have frequent presence at the household level, through both announced and unannounced household visits to assess technology adoption involving local officials and other local stakeholders.
- Community Hygiene Clubs – Organized activities address community hygiene clubs specifically with benefits and ask members to advocate *Tubeho Neza* products.

DelAgua staff are also responsible for repair and replacement of technologies. Reporting of broken products initiated by households or community leaders calling staff directly or the DelAgua cellular hotline. Each report is documented and assigned. Staff are then responsible for performing community based repairs or replacements in areas where they are needed, which are reported when completed and tracked in a Work Order system. Replacement parts are stored at the District and Sector level to provide easy access for staff.

### Survey methods

Two types of survey data are described throughout this study; those collected by CHWs on nearly 100 % of all households and data from a verification survey (VS) administered to a sample of the households. Throughout the results section, these surveys will be referred to as “CHW” or “VS” respectively to distinguish the origin of the data.

All surveys were tracked through electronic forms sent to the DelAgua server, hosted by doForms, Inc., a smartphone based, online hosted survey provider. An online dashboard tracked the number of forms received against expected target numbers. Additional analysis was completed on a dashboard to identify duplicate or abnormal forms, which could then be relayed to field staff for arbitration. Data was then analyzed using R-Project, a open source statistical software. Any missing data was excluded from the analysis and any outlier exclusion is noted in the analysis.

### CHW surveys

Data was collected by CHWs during three distinct activities; during the distribution, at the initial household visit approximately a day to a week after distribution, and the follow up visit conducted approximately 6 weeks to 6 months after distribution. Metrics addressed in each survey are described throughout the previous section. All CHW surveys were conducted on 100 % of households unless households could not be found. The survey portion of a CHWs household visit.

### Verification surveys

Two rounds of detailed verification surveys were conducted; one between January and April of 2015, approximately 6 weeks to 6 months after distribution, and the second between July and September of 2015, approximately 10 months to a year after distribution. The surveys were designed to provide programmatic information to the implementer while at the same time satisfying the verification requirements for carbon credits. The surveys were administered by DelAgua staff and included parameters required by the UN Clean Development Mechanism (CDM) monitoring guidelines and methodologies [13]. The CDM also requires a third party auditor to verify survey data and perform field visits to a sample of surveyed households. Additional guidance used by the implementer for programmatic data included a World Health Organization manual on monitoring and evaluation for household water treatment programs [14]. Additional questions were included to assess environmental, health, social and livelihood benefits of the program. The survey instrument consisted of over 100 questions and took approximately 45 min to an hour to administer. Households were provided one kilogram of rice and one liter of cooking oil for participating in the survey. It was piloted extensively and enumerators were required to attend a 3 day training on administration of the survey including field based practice surveys in households.

The sampling strategy differed in each survey round. For the first verification survey, a two-stage, cluster sample design was employed. In the first stage, 320 villages in Western Province were randomly selected with probability proportionate to size (PPS) sampling (the number of recipient households was used as the measure of size). In the second stage, three households within each village were randomly selected using simple random sampling (SRS). This resulted in a self-weighted sample of 960 households. At the end of the sampling period, an additional 40 households were selected using SRS and added to the sample to meet CDM requirements, bringing the total number of surveyed households to 1000. During the second verification, only a simple random sample was used, for 187 valid surveys. Household that could not be found, did not consent or did not have an adult over the age of 18 responding were not surveyed and the next household in the randomly generated list was visited. To avoid a potential source of survey bias, surveyors were not provided with this list in advance, and instead contacted the survey manager for the next house on the list when necessary.

### Ethics and consent

The Rwanda National Ethics Committee (IRB #197/RNEC/2014) approved the protocol including all questions and the consent procedure for all CHW surveys and the verification survey. Each household gave informed, verbal consent after receiving details regarding the purpose of the survey. All respondents had to additionally be over the age of 18. Verbal consent was requested and approved based on the high percentage of illiteracy within the study population. Consent was administered through the smartphone survey with all records stored on a password protected server. Participants were given the opportunity to ask questions before consenting to participate. Additionally all households, regardless of consenting to the surveys were able to retain the filter and cookstove.

## Results

### Product delivery

A total of 457,778 people across 101,778 households received water filters and cookstoves during the initial campaign distribution. Of these households, 88 % (89,609) were households classified as *Ubudehe* 1 or 2 with the remaining 12 % (12,157) consisting of households from local cell and village officials, local community health workers and pilot households outside of the *Ubudehe* 1 and 2 classification (Table 1). Following the distribution, community health workers visited 97.8 % (99,515) of households to perform household level education and training activities. Average household size was 4.5 with 0.61 children under five. Before receiving the water filter and cookstove, 89.0 % households reported firewood as their primary fuel source with three quarters (76.1 %) of households reporting the traditional three stone fire as their primary cookstove and the majority (59.2 %) reporting primarily cooking indoors. Most households reported the public tap (43.6 %) or protected spring (31.1 %) as their primary water source with a quarter (26.6 %) reporting treating their water before receiving the filter mostly by boiling (80.7 % of households reporting treating their water) (Table 2). While boiling can be an effective treatment method, meeting WHO standards for microbial contamination, it is unclear how consistently or sufficiently water boiling households engaged in the practice.

**Table 1:** Program Delivery

	Product Distribution	Initial Household Education Visit		Follow up Household Visit	
	*n*	*n*	% of distribution	*n*	% of distribution
Households Reached	101,778	99,515	97.8 %	98,804	97.1 %
Ubudehe 1 or 2 Households	89,609	87,728	97.9 %	86,859	96.9 %
Households Outside of Ubudehe 1 or 2	12,157	11,787	97.0 %	11,945	98.3 %
Total Beneficiaries	457,778	451,236	98.6 %	449,882	98.3 %

**Table 2:** Baseline Metrics

	*n*	%	95 % CI
Household Size	4.5 (SD: 2.1)		
Children Under 5	0.61 (SD: 0.89)		
Baseline Cooking Location			
Indoor	57553	59.2 %	0.31 %
Outdoor	7910	8.1 %	0.17 %
Separate Kitchen	31627	32.5 %	0.29 %
Other	125	0.1 %	0.02 %
Baseline Primary Stove			
Traditional 3-Stone Fire	75690	76.1 %	0.27 %
Rondereza	19564	19.7 %	0.25 %
Imbabura	3176	3.2 %	0.11 %
Other	1058	1.1 %	0.06 %
Additional Baseline Stoves			
Traditional 3-Stone Fire	75070	75.3 %	0.27 %
Rondereza	19053	19.1 %	0.24 %
Imbabura	3406	3.4 %	0.11 %
Other	2195	2.2 %	0.09 %
Baseline Fuel			
Wood	88583	89.0 %	0.19 %
Straw/Shrubs/Grass	7124	7.2 %	0.16 %
Agricultural Crop	283	0.3 %	0.03 %
Charcoal	3159	3.2 %	0.11 %
LPG/Natural Gas/Biogas	331	0.3 %	0.04 %
Other	35	0.0 %	0.01 %
Primary Water Source			
Public Tap	43389	43.6 %	0.31 %
Protected Spring	30935	31.1 %	0.29 %
Unprotected Spring	10627	10.7 %	0.19 %
Handpump	4037	4.1 %	0.12 %
River	3648	3.7 %	0.12 %
Protected Dug Well	2359	2.4 %	0.09 %
Piped in Home or Compound	1367	1.4 %	0.07 %
Unprotected Dug Well	1341	1.3 %	0.07 %
Lake	1061	1.1 %	0.06 %
Other	682	0.7 %	0.05 %
Baseline Treating Water	26432	26.6 %	0.27 %
Baseline Water Treatment Method			
Boiling	21329	80.7 %	0.48 %
Sur Eau	4295	16.2 %	0.44 %
Other	808	3.1 %	0.21 %

Overall, 90 % of households identified on the *Ubudehe* list received products. Most households not reached on the *Ubudehe* list were attributed to discrepancies such as households listed multiple times or households which had moved out of the intervention area. Over the course of the initial campaign, 212 (0.2 %) products were repossessed for reasons including allocation to the incorrect household (119, 0.1 %), a household receiving multiple products (59, 0.1 %) or a household selling their filter or cookstove (17, 0.02 %).

The follow up campaign reached 98,804 (97.1 %) of the households that were originally distributed technologies. CHWs recorded just over 1 % of stoves missing (1164, 1.2 %) and under 1 % of filters missing (930, 0.9 %) during the follow up household visits. Missing products were primarily attributed to stolen products (335 (0.3 %) stoves, 138 (0.1 %) filters), sold products (315 (0.3 %) stoves, 261 (0.3 %) filters), products being kept at a relative or neighbor’s house (263 (0.3 %) stoves, 208 (0.2 %) filters) and products being stored in a locked room where the CHW could not confirm the presence of the products at the time of the visit (210 (0.2 %) stoves, 254 (0.3 %) filters). Only minor hardware issues with the stoves were reported by CHWs, and these did not require replacement or repair. However, CHWs performed about 1500 repairs to filters (1460, 1.5 %) which primarily consisted of unclogging filters through multiple backwashes (590, 0.6 %), reassembling leaking filters (567, 0.6 %) and replacing defective or missing parts (252, 0.3 %) such as plastic tubing, o-rings, taps, backwashing tanks and pre-filters.

Since the follow up campaign, DelAgua staff have continued to perform repair and replacement activities throughout the intervention area. Approximately 12 months following the original distribution, stoves required minimal maintenance. Filters have required more attention with 187 (0.2 %) filter replacements primarily from households trying to disassemble the filters with staff finding either the water nozzle (83, 0.1 %) or plastic joint connecting the dirty water and safe storage sides of the filter (36, 0.04 %) broken. Additionally 931 (0.9 %) filter repairs have been performed, mostly attributable to the replacement of the backwashing tube (649, 0.7 %) which is more vulnerable to damage because it is the only exposed soft-goods portion of the filter. Other filter repairs included backwashing clogged filters (117, 0.1 %) and the reassembling of the joint between the filter (26, 0.03 %) when it did not require a full replacement.

### Social marketing activities

Households participating in the verification survey reported first hearing about the program through local officials (38.9 %), the initial distribution meeting (20.8 %) or their local CHW (14.9 %). The two targeted social marketing activities, sensitization meetings and radio advertisements, were not widely reported as the initial pathway for program awareness with just 9.2 % and 1.9 % respectively of households reporting as their first exposure to the program. However, over three quarters (75.7 %) of households did report attending the sensitization meetings while only a quarter of households (23.2 %) reported hearing any of the radio advertisements.

### Water filter adoption indicators

Tables 3 and Additional file 1: Table S2 detail water filter adoption indicators, including values described below. The CHW follow up survey of the majority of households and the two more comprehensive verification survey rounds of a subset of the households, all measured the reported filter adoption above 90 % and observed filter adoption above 75 %. During the CHW follow up visits, 94.1 % of households confirmed treating the last water they consumed with 99.5 % of those households reporting using the LifeStraw filter as the water treatment method (93.6 % filter adoption population-wide). The first verification survey conducted concurrently with the CHW follow up survey, measured 95.9 % treating the last water and again 99.5 % reporting the filter as the treatment method (95.4 % filter adoption including non-treaters). The second verification, performed at least 10 months after distribution showed a small decrease in adoption with 92.0 % of households reporting treating the last water they consumed and 99.4 % reporting the filter as the treatment method (91.4 % filter adoption including non-treaters). Observed filter adoption, measured by water present in the filter at the time of the visit, was observed in 78.7 % of households visited by CHWs, 81.1 % of households during the first verification round and then a decrease of nearly 5 % (76.5 %) in the second verification round (Table 3).

**Table 3:** Water Filter Adoption Indicators

	CHW Follow Up Survey			Verification Round 1–6 weeks to 6 months after distribution			Verification Round 2–10 months to 1 year after Distribution		
	*n*	%	95 % CI	*n* or value	%	±95 % CI	*n* or value	%	±95 % CI
Filter Present	97874	99.1 %	0.06 %	996	99.6 %	0.39 %	185	98.9 %	1.47 %
Reported Treating Last Water Consumed	92940	94.1 %	0.15 %	959	95.9 %	1.23 %	172	92.0 %	3.89 %
Reported Last Water Treatment Method									
LifeStraw Filter	92438	93.6 %	0.15 %	954	95.4 %	1.30 %	171	91.4 %	4.01 %
Boiling	466	0.5 %	0.04 %	4	0.4 %	0.39 %	1	0.5 %	1.05 %
Other	3	0.003 %	0.00 %	1	0.1 %	0.20 %	0	0.0 %	0.00 %
Water Present in Filter	77790	78.7 %	0.26 %	811	81.1 %	2.43 %	143	76.5 %	6.08 %
Reported Ever Drinking Untreated Water at Home				26	2.7 %	1.00 %	7	4.0 %	2.79 %
Reported Ever Drinking Untreated Water Away from Home				300	31.0 %	2.87 %	36	20.3 %	5.77 %
Reported Location Drinking Untreated Water Away from Home									
While Traveling				160	34.7 %	2.95 %	22	41.5 %	7.06 %
School				134	29.1 %	2.81 %	16	30.2 %	6.58 %
Work				130	28.2 %	2.79 %	13	24.5 %	6.17 %
Don’t Know				20	4.3 %	1.26 %	1	1.9 %	1.95 %
Other				17	3.7 %	1.17 %	1	1.9 %	1.95 %
Reported Filtered Water Quantity (lppd) - Inclusive of Non-Users				1.64 (SD: 1.21)			1.63 (SD: 1.24)		
Reported Storing Filtered Water				663	68.5 %	2.88 %	114	64.4 %	6.86 %
Storage Vessel									0.00 %
Covered Container with Lid				551	80.3 %	2.46 %	108	93.9 %	3.43 %
Uncovered Container				118	17.2 %	2.34 %	5	4.3 %	2.92 %
Other				12	1.7 %	0.81 %	2	1.7 %	1.87 %

Additional questions were asked of verification survey households only. During both rounds, over 80 % of households reported filling the filter today (44.8 % - 1^st^ VS, 41.8 % - 2^nd^ VS) or yesterday (42.8 % -1^st^ VS, 44.6 % - 2^nd^ VS) with the remainder (12.4 % - 1^st^ VS, 13.6 % - 2^nd^ VS) reporting filtering more than 2 days ago or not knowing the last time the filter was filled. Additionally households were asked to demonstrate use of the filter. Enumerators recorded performance in meeting up to seven actions. Most households in both rounds (97.5 % - 1^st^ VS, 97.3 % - 2^nd^ VS) were given a rating of sufficient or higher, with nearly 50 % (48.9 %, 43.8 %) receiving excellent ratings. Only 25 households in the 1^st^ round and 5 households in the second round (2.5 % - 1^st^ VS, 2.7 % - 2^nd^ VS) were given a rating of insufficient and thus unable to demonstrate proper usage of the filter.

Households who did not report treating their water during either verification survey round (56 households total), reported this was due to habit (26.9 %), their filter being damaged (16.4 %) and no availability of water in the home (13.4 %). While the 6 verification households who reported using a different treatment method, did so because their filter wasn’t working (36.4 %) and they didn’t know how to use the filter (27.3 %).

Extensive piloting was conducted to determine the likely least subjective method of determining water volume treated. Quantity of water treated was calculated by the size of the vessel reported used to fill the filter multiplied by the reported number of times the filter was filled each day. This was divided by the number of persons (adults and children) living in the household to yield the liters per person per day (LPPD). Average filtered water volume across the sample, including non-users (0 l per day) was 1.48 (SD = .80) liters per person per day during the first round and 1.44 (SD = .72) liters per person per day during the second round. The majority of households (81.9 % - 1^st^ VS, 84.2 % - 2^nd^ VS) use filtered water only for consumption with the remaining households (18.1 % - 1^st^ VS, 15.8 % - 2^nd^ VS) using filtered water for additional purposes including cleaning the filter (40.9 % - all VS), washing dishes (29.6 % - all VS) and cooking (18.7 % - all VS). Households reported a 140 % increase in the first round (SD: 139 %) and a 161 % increase in consumption of water from before receiving the filter to after.

**Fig. 3:**
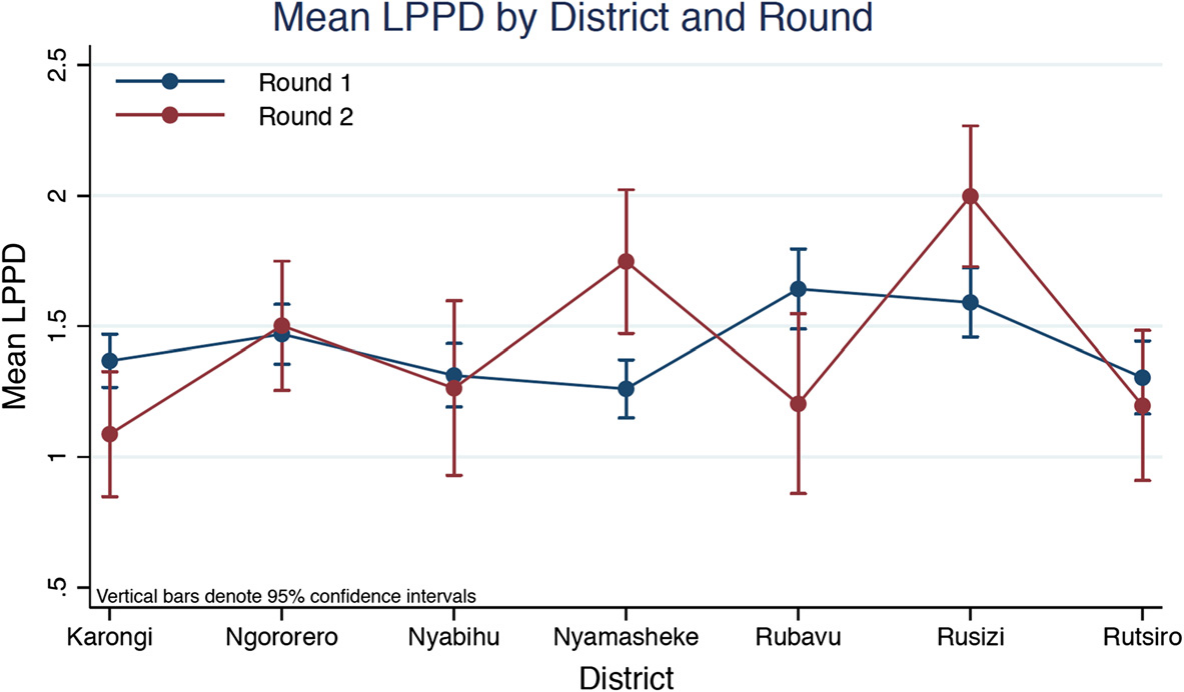
Mean reported filtered water consumed per person per day by district and verification survey round

Differences in household water filter use between the seven districts in the Western Province and across verification survey rounds were assessed using linear regression with robust standard errors. We observed significant differences in mean LPPD between districts in both round 1 (*p* < .001) and round 2 (*p* < .001). During round 1, mean LPPD ranged from 1.28 (95 % CI = 1.16–1.39) in Nyamasheke to 1.71 (95 % CI = 1.56–1.86) in Rusizi. During round 2, mean LPPD ranged from 1.09 (95 % CI = .84–1.34) in Karongi to 1.99 (95 % CI = 1.83–2.17) in Rusizi. While there was no overall difference in LPPD between round 1 and round 2, there were statistically significant increases in the districts of Nyamasheke (Δ = .47, 95 % CI = .18–.76, *p* = .001) and Rusizi (Δ = .29, 95 % CI = .06–.51, *p* = .013) and decreases in the districts of Karongi (Δ = −.35, 95 % CI = −.62 to −.08, *p* = .012) and Rubavu (Δ = −.56, 95 % CI = −.84 to −.28, *p* < .001) (Fig. 3).

Drinking untreated water was reported in 369 of verification survey responses with 33 (2.9 %) households reporting drinking some untreated water at home and 336 (29.3 %) households reporting drinking some untreated water away from home. When drinking water outside of the home, households were primarily traveling (35.4 %), at school (29.2 %) or at work (27.8 %).

While the filter itself has approximately 5.5 l of storage capacity, 67.8 % of households across both verification rounds report storing additional filtered water. The majority (82.8 %) store in a covered container which is usually a jerry can of various sizes. Households who store water report cleaning their storage container at least once a week (96.8 %) mostly with filtered water (44.9 %) and untreated water (24.2 %). Additionally the safe storage symbol which was promoted through the program to be affixed to any storage containers designated for safe water storage was observed on 89.3 % of containers identified as water storage containers by households.

The primary maintenance task required for the filter is backwashing of the filter membrane. Most verification households (95.5 %) reported backwashing their filter every time they filtered water as advised during household education. However, when asked to demonstrate use of their water filter, only about half of households demonstrated backwashing and safe disposal of backwashed water.

Additional findings include that many (70.9 %) households in the verification sample share water with people outside their household. Of the households that shared water, only 19.7 % reported usually sharing, while the remaining 80.3 % reported sharing sometimes or rarely. Verification households generally did not have negative feedback on how to improve the filter, with most households (69.4 %) reporting no changes to the filter. Other responses included increasing the volume (8.9 %), adding a stand to the bottom of the filter (5.8 %) and providing a cleaning accessory for easier maintenance (4.9 %). Additionally households primarily reported that they liked the filter because it provided clean water (43.7 %), they like the taste of the water (14.2 %), it provides safe water storage (10.6 %) and it saves fuelwood from not having to boil water (10.3 %).

### Improved cookstove adoption indicators

Tables 4 and Additional file 1: Table S3 detail water filter adoption indicators, including values described below. 92.8 % of households in both the CHW survey (91,704 of 98,804) and the first verification survey (928 of 1000) reported the EcoZoom stove as their primary cookstove, with a small decrease to 89.3 % during the second verification round. The next most frequent response was the traditional three stone fire with less than 5 % for the CHW survey and the first verification round (4.9 %) with an increase to 9.6 % during the second verification round. When asked which stove was cooked on during the last cooking event, EcoZoom use reduced to around 80 % of responses (79.2 % CHW, 82.0 % 1^st^ VS, 80.5 % 2^nd^ VS) while the traditional three stone fire increased by less than 15 % for all rounds (14.6 %). Observed EcoZoom use was also lower based on stoves that CHWs and enumerators witnessed cooking on at the time of the household visit (75.2 % CHW, 77.9 % 1^st^ VS, 83.3 % 2^nd^ VS). Additionally, households reported use of the pot skirt, in about 7 out of 10 cooking events during both verification rounds (68.9 % 1^st^ VS, 67.1 % 2^nd^ VS) (Table 4).

**Table 4:** Improved Cookstove Adoption Indicators

	CHW Follow Up Survey			Verification Round 1–6 weeks to 6 months after distribution			Verification Round 2–10 months to 1 year after Distribution		
	*n*	%	95 % CI	n or value	%	±95 % CI	*n* or value	%	±95 % CI
EcoZoom Present	97640	98.8 %	0.07 %	996	99.6 %	0.39 %	186	99.5 %	1.05 %
Stove Type - Cooking at Time of Visit	14358	14.7 %	0.22 %	181	18.2 %	2.39 %	30	16.1 %	5.27 %
EcoZoom	10798	75.2 %	0.27 %	144	77.8 %	2.57 %	25	83.3 %	5.34 %
Traditional 3-Stone Fire	2374	16.5 %	0.23 %	20	10.8 %	1.92 %	6	20.0 %	5.73 %
*Rondereza* - Locally Made Wood Burning Stove	864	6.0 %	0.15 %	15	8.1 %	1.69 %	3	10.0 %	4.30 %
*Imbabura* - Locally Made Charcoal Stove	200	1.4 %	0.07 %	6	3.2 %	1.10 %	0	0.0 %	0.00 %
Reported Last Time Cooking Stove									
EcoZoom	80954	79.2 %	0.25 %	838	82.0 %	2.38 %	157	80.5 %	5.68 %
Traditional 3-Stone Fire	14942	14.6 %	0.22 %	118	11.5 %	1.98 %	27	13.8 %	4.95 %
*Rondereza* - Locally Made Wood Burning Stove	4877	4.8 %	0.13 %	53	5.2 %	1.37 %	7	3.6 %	2.67 %
*Imbabura* - Locally Made Charcoal Stove	936	0.9 %	0.06 %	12	1.2 %	0.67 %	4	2.1 %	2.03 %
Other	482	0.5 %	0.04 %	1	0.1 %	0.19 %	0	0.0 %	0.00 %
Reported Primary Stove			0.00 %						
EcoZoom	91704	92.8 %	0.16 %	928	92.8 %	1.60 %	167	89.3 %	4.43 %
Traditional 3-Stone Fire	4829	4.9 %	0.13 %	49	4.9 %	1.34 %	18	9.6 %	4.23 %
*Rondereza* - Locally Made Wood Burning Stove	1711	1.7 %	0.08 %	17	1.7 %	0.80 %	0	0.0 %	0.00 %
Imbabura - Locally Made Charcoal Stove	436	0.4 %	0.04 %	5	0.5 %	0.44 %	2	1.1 %	1.47 %
Other	124	0.1 %	0.02 %	1	0.1 %	0.20 %	0	0.0 %	0.00 %
Reported Use of Other Stoves Besides Primary Stove	48013	48.6 %	0.31 %	475	47.5 %	3.10 %	96	51.3 %	7.16 %
Reported Type of Stoves Used Other than Primary Stove									
EcoZoom				63	12.5 %	2.05 %	20	19.8 %	5.71 %
Traditional 3-Stone Fire				277	55.2 %	3.08 %	53	52.5 %	7.16 %
*Rondereza* - Locally Made Wood Burning Stove				112	22.3 %	2.58 %	18	17.8 %	5.49 %
*Imbabura* - Locally Made Charcoal Stove				49	9.8 %	1.84 %	10	9.9 %	4.28 %
Other				1	0.2 %	0.28 %	0	0.0 %	0.00 %
% Of Cooking Events on EcoZoom Stove					86.4 % (SD: 18.4 %)			92.5 % (SD:12.7 %)	
Location - Cooking at Time of Visit						0.00 %			
Indoor				31	16.8 %	2.31 %	3	9.7 %	4.24 %
Outdoor with Cover				14	7.6 %	1.64 %	0	0.0 %	0.00 %
Outdoor without Cover				102	55.1 %	3.08 %	19	61.3 %	6.98 %
Doorway				17	9.2 %	1.79 %	2	6.5 %	3.52 %
Separate Kitchen				21	11.4 %	1.97 %	7	22.6 %	5.99 %
Reported Primary Cooking Location									
Indoor	6427	6.5 %	0.15 %	60	6.0 %	1.47 %	22	11.8 %	4.62 %
Outdoor with Cover	4668	4.7 %	0.13 %	69	6.9 %	1.57 %	7	3.7 %	2.72 %
Outdoor without Cover	60548	61.3 %	0.30 %	695	69.5 %	2.85 %	134	71.7 %	6.46 %
Doorway	21259	21.5 %	0.26 %	115	11.5 %	1.98 %	11	5.9 %	3.37 %
Separate Kitchen	5835	5.9 %	0.15 %	59	5.9 %	1.46 %	13	7.0 %	3.65 %
Other	67	0.1 %	0.02 %	2	0.2 %	0.28 %	0	0.0 %	0.00 %
Reported Fewer Cooking Events Per Week Indoors				7.33 (SD: 5.87)			7.23 (SD: 4.61)		
Fuel - Cooking at Time of Visit									
Wood				166	89.2 %	1.92 %	29	93.5 %	3.52 %
Straw/Shrubs/Grass				11	5.9 %	1.46 %	2	6.5 %	3.52 %
Agricultural Crop				1	0.5 %	0.45 %	0	0.0 %	0.00 %
Charcoal				7	3.8 %	1.18 %	0	0.0 %	0.00 %
LPG/Natural Gas/Biogas				0	0.0 %	0.00 %	0	0.0 %	0.00 %
Electricity				0	0.0 %	0.00 %	0	0.0 %	0.00 %
Other				1	0.5 %	0.45 %	0	0.0 %	0.00 %
Reported Primary Cooking Fuel									
Wood	95864	97.0 %	0.11 %	970	97.0 %	1.06 %	181	96.8 %	2.53 %
Straw/Shrubs/Grass	2343	2.4 %	0.09 %	17	1.7 %	0.80 %	4	2.1 %	2.07 %
Agricultural Crop	170	0.2 %	0.03 %	3	0.3 %	0.34 %	0	0.0 %	0.00 %
Charcoal	334	0.3 %	0.04 %	6	0.6 %	0.48 %	1	0.5 %	1.05 %
LPG/Natural Gas/Biogas	47	0.0 %	0.01 %	0	0.0 %	0.00 %	0	0.0 %	0.00 %
Electricity	12	0.0 %	0.01 %	0	0.0 %	0.00 %	0	0.0 %	0.00 %
Other	34	0.0 %	0.01 %	4	0.4 %	0.39 %	1	0.5 %	1.05 %

The 10 households (0.8 %) between both verification survey rounds which reported not using the EcoZoom stove, reported they didn’t know how to use it (23.1 %), it didn’t warm the house (23.1 %) or it was difficult to use (15.4 %) as the reported reasons for non-use.

Enumerators performing the verification survey asked households to demonstrate proper cookstove use with each household receiving an internally recorded rating based on number of successful use and maintenance steps completed. Almost all households (98.3 %) received a rating of sufficient to use the EcoZoom stove or better with 79.0 % of households receiving an excellent rating. Only 1.7 % of households received a rating of insufficient for use of the cookstove.

While households reported use of a primary stove, about half the households (48.6 % CHW, 47.5 % 1^st^ VS, 51.3 % 2^nd^ VS) reported usage of other stoves as well. The traditional three stone fire (54.7 % all VS) was the most common supplementary stove followed by the *Rondereza* (21.6 % all VS). Based on the number of cooking events reported by each verification household, the EcoZoom was used on average in 86.4 % (SD: 18.4 %) of a household’s cooking events during the first verification round and then increased to 92.5 % during the 2^nd^ verification round. The most frequently reported reasons for using another stove included difficulty in finding dry fuelwood to use in the EcoZoom stove (32.2 %), the need to use multiple stoves at one time (24.2 %) and the need to warm the home (15.1 %).

**Fig. 4:**
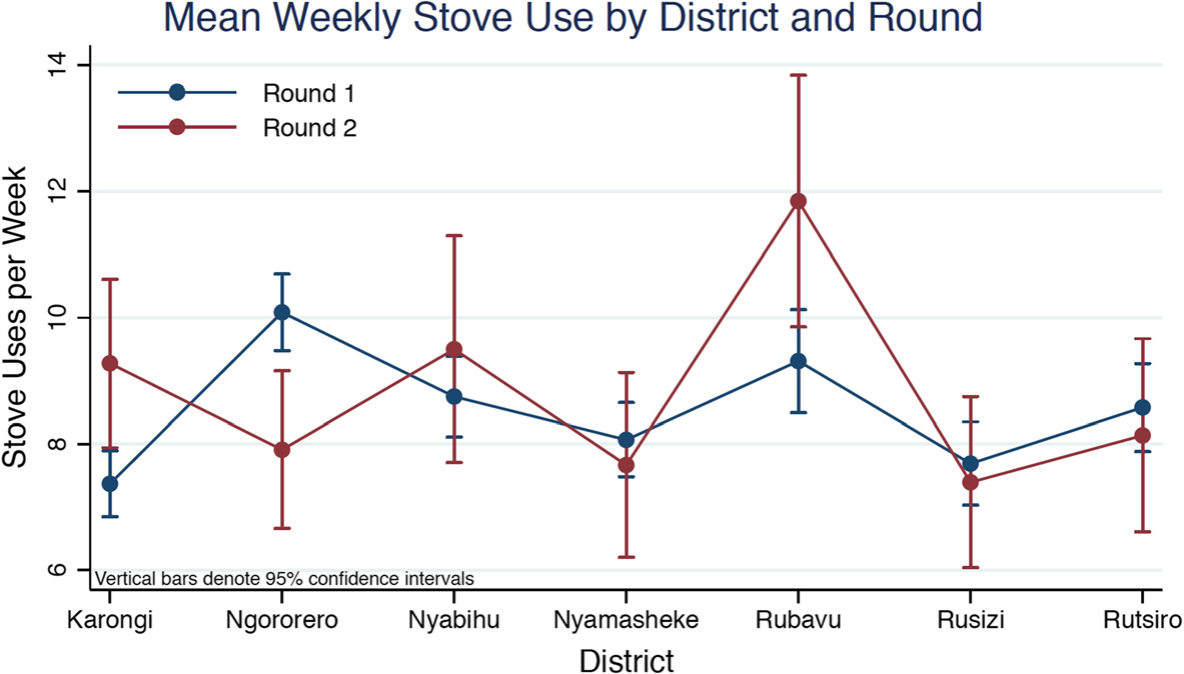
Mean reported stove uses per week by district and verification survey round

Reported weekly Ecozoom stove use was compared between the seven districts in the Western Province and between survey rounds using Poisson regression with robust standard errors. Significant differences in the count of weekly household Ecozoom uses was observed between districts (*p* < .001) (Fig. 4). The average weekly number of EcoZoom uses reported during round 1 ranged from 7.50 (95 % CI = 6.95–8.05) in Karongi to 10.15 (95 % CI = 9.51–10.79) in Ngororero. During round 2, weekly use ranged from 7.40 (95 % CI = 6.69–8.10) in Rusizi to 12.09 (95 % CI = 10.81–13.36) in Rubavu. There was a significant increase in EcoZoom use from round 1 to round 2 in Karongi (Δ = 2.73, 95 % CI = 1.34–4.12, *p* < .001) and Rubavu (Δ = 2.64, 95 % CI = 1.05–4.23, *p* = .001), and a significant decrease in Ngororero (Δ = -2.35, 95 % CI = −3.45 to −1.24, *p* < .001).

**Fig. 5:**
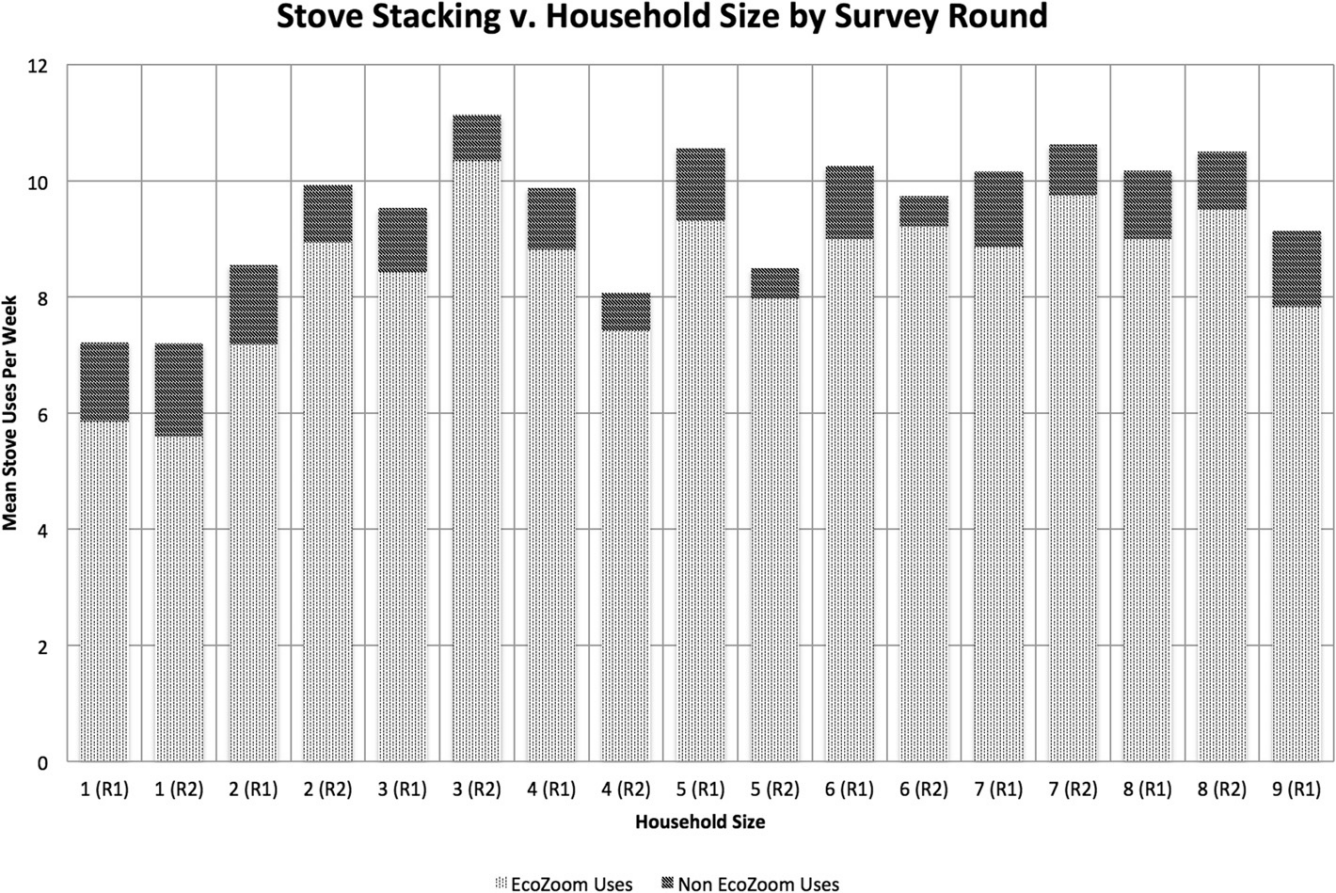
Mean reported improved stove used and traditional stove use per week by household size and verification survey round

To evaluate if stove stacking behavior corresponded to larger household size, the relationship between household size and the weekly count of both baseline (traditional) and EcoZoom stove use was examined using Poisson regression with robust standard errors. We found no significant association between the number of traditional stove uses and household size in either survey round 1 (IRR = 1.00, 95 % CI = .96–1.05, *p* = .914) or round 2 (IRR = .94, 95 % CI = .79–1.11, *p* = .457). The mean number of weekly EcoZoom and traditional stove uses by household size and survey round are shown in Fig. 5.

Wood was the primary reported cooking fuel in about 97 % of households for all surveys (97.0 % CHW survey, 97.0 % 1^st^ VS, 96.8 % 2^nd^ VS), though only 90.0 % of households were using wood in observed cooking events by verification survey enumerators. Most verification households reported only collecting wood (74.1 %) while 10.2 % reported both collecting and purchasing wood, and the remainder (15.7 %) only purchasing wood. 92.8 % of households reported storing wood, a highly emphasized part of the education program to promote drying of wet fuelwood, with most households storing wood inside the home (59.7 %) and a third storing in a separate kitchen (34.4 %).

The majority of households reported cooking outdoors (66.0 % CHW, 76.4 % 1^st^ VS, 75.4 % 2^nd^ VS) with cooking in a doorway (21.5 % CHW survey, 11.5 % 1^st^ VS, 5.9 % 2^nd^ VS) as the next most frequent cooking location. Slightly lower outdoor cooking (62.7 % 1^st^ VS, 61.3 % 2^nd^ VS) was observed when households were cooking at the time of the verification household visits with over a quarter (28.1 % 1^st^ VS, 32.3 % 2^nd^ VS) of households cooking indoors or in a separate kitchen. Households reported cooking indoors fewer times per week than before receiving the EcoZoom stove (7.33 1^st^ VS, 7.23 2^nd^ VS). Primarily households reported cooking indoors because they were getting away from rain (33.8 %) followed by cooking on a stove that could not be moved outdoors (18.7 %), the need to warm the house (12.3 %), security (9.7 %) and habit (9.6 %).

When asked what could be improved on the stove, the majority of verification household’s responses were no improvements (60.4 %) with other frequent responses including increasing the size of the stick support (11.9 %), increasing the size of the stove top (7.8 %) and providing a stove that can use multiple fuels (7.1 %). Households additionally reported liking the stove because it cooks fast (32.9 %), reduces fuelwood (30.5 %) and produces less smoke (19.9 %).

### Quality assurance evaluation

To reinforce the value of household education and interaction, several quality assurance activities were instituted. Before CHWs were allowed to perform household visits alone, a group household visit was conducted with the supervisor to offer feedback and provide clarification for a high quality household visit. CHWs were continually tracked against several metrics including number of surveys per day, average time spent in households and a qualitative evaluation performed by their supervisor. Of 864 CHWs, 774 (89.6 %) evaluations were submitted by DelAgua staff. About a tenth (10.9 %) of CHWs received an excellent rating, three quarters (74.5 %) received a satisfactory rating and the remainder (14.6 %) received an unsatisfactory rating. CHW performance during household visits was evaluated by number of surveys, average survey time and an additional qualitative evaluation performed by staff during one of the CHWs first visits. On average CHWs performed seven household surveys per day, spending 31 min in a household. CHW evaluations improved slightly from the refresher training with under a tenth (9.1 %) of CHWs performing to an unsatisfactory rating, just over 80 % (80.9 %) receiving a satisfactory rating and 10.0 % receiving an excellent rating (Table 5). Some CHWs receiving unsatisfactory ratings were dismissed.

**Table 5:** CHW Quality Control Indicators

	Refresher Training	Product Distribution	Initial Household Education Visit	Follow up Household Visit
Total CHWs		856	849	820
CHWs per day		71 (31–132) SD: 15	208 (139–309) SD: 60	444 (41–604) SD: 162
Surveys Per Day		1094 (1–463) SD: 943	1037 (9–2274) SD: 659	1176 (3–3557) SD: 1237
Surveys Completed per CHW		119 (1–714) SD: 61	117 (1–259) SD: 41	120 (1–226) SD: 39
Surveys Completed per CHW per day		24 (6–71) SD: 8	7 (2–12) SD: 1	5 (2–6) SD: 1
CHW Survey Time (minutes)		Not Collected	31 (1–119) SD: 14^a^	46 (1–119) SD: 19^a^
CHW Evaluations				
Excellent	84 (10.9 %)		72 (10.0 %)	571 (71.6 %)
Satisfactory	577 (74.5 %)		585 (80.9 %)	224 (28.1 %)
Unsatisfactory	113 (14.6 %)		66 (9.1 %)	3 (0.3 %)

^a^Surveys greater than 2 h were discounted as outliers

CHW metrics were again tracked during the follow up household visits, including supervisor evaluations of CHW education performance through visiting households previously visited by CHWs. Supervisors evaluated a CHWs completion of all education tasks including the presence of the hung poster, the sticker placed on an appropriate safe storage container, and bracelets given to households for adopting the products. Additionally households were asked several questions related to retention of key messages and asked to demonstrate use. A score was calculated based on these metrics and CHWs were ranked as excellent (71.6 %), satisfactory (28.1 %) or unsatisfactory (0.3 %) performers. High performing CHWs were given a bonus, satisfactory CHWs were given no bonus, and unsatisfactory performers were reviewed further for dismissal from the program. Evaluated households were selected by the supervisors with CHW’s having no prior knowledge as to which specific household might be selected. On average CHWs performed five household visits per day, slightly lower than the initial household survey of seven per day due to the longer time spent in households (46 min).

## Discussion and conclusions

During a 3-month period in late 2014, over 470,000 people living in over 101,000 households were provided free water filters and cookstoves. Approximately a year after distribution, reported water filter use was above 90 % (+/−4 % CI) and water present in filter was observed in over 76 % (+/−6 % CI) of households, while the reported primary stove was nearly 90 % (+/−4.4 % CI) and of households cooking at the time of the visit, over 83 % (+/−5.3 % CI) were on the improved stove.

### Program implementation

The extensive process of updating the *Ubudehe* distribution list before distribution proved essential with over 90 % of households accurately distributed products. Reaching each individual household for education and training proved to be challenging. CHWs often had to travel many hours to reach target households and thus a large proportion of time and resources was spent on finding the last few households in each village. Local officials and CHWs were helpful in identifying and finding missing households and only about 2 % were unaccounted for during the first household visits and 3 % during the following up campaign months after the distribution.

Only 21 (0.02 %) products were repossessed during the initial campaign due to a product being sold or stolen and 650 (0.65 %) products during the months of the follow up campaign (Additional file 1: Table S1). Possibly contributing to these low rates are the products marked as “not for sale” and a signed agreement between the household and a local official which outlined the use and benefits of the technologies for only the household who received the technologies. The considerable support of Rwandan government officials in stressing to households the importance of the technologies as well as the already established programs which offer free services to *Ubudehe* 1 and 2 households could be additional contributors.

Social marketing is often promoted as an important strategy in behavior change programs [15]. The *Tubeho Neza* program employed radio advertisements and sensitization meetings as social marketing mechanisms to raise program awareness and provide initial knowledge to households. The use of mass media such as radio advertisements has been used in several water and sanitation interventions [15], however the verification survey only measured a quarter of households ever hearing the radio advertisements and less than 2 % identified it as their initial exposure to the program. Similarly only a fraction of households identified the sensitization meeting as their first communication about the program, but many households did report attending the sensitization meeting. Additionally many program staff reported the importance of the sensitization meetings because of the initial exposure of households to the technologies before receiving them. Households were perceived to be more comfortable with initial usage of the products during distribution because of the knowledge gained from the sensitization meetings. Still, the most frequent response to initially hearing about the *Tubeho Neza* program was through local officials, suggesting dissemination of information can effectively be done through already established government programs in Rwanda.

CHWs were an integral part of reaching beneficiaries at the household level and providing quality education and training. Past CHW based programs have shown varied results to the effectiveness of CHWs with evidence suggesting poor performance for a variety of reasons from poor selection of CHWs to low levels of training to lack of on-going support and supervision [16]. The *Tubeho Neza* program sought to mitigate many of these downfalls through an extensive selection and training program paired with an interactive household education platform that was closely evaluated and monitored by program staff. Performance metrics from number of surveys completed to survey time to qualitative evaluations revealed that most CHWs were performing to at least a satisfactory if not excellent level and CHW metrics improved from the initial campaign to the follow up visits. Still, CHW performance was varied as revealed by evaluations from the program staff. One common issue that arose late in the campaign was the time between the initial trainings and CHW teams which started later in the campaign. Some teams did not begin program activities until a couple of months after the District level trainings and low retention of some concepts was noticed. Further training and continued tracking of CHW performance are essential in providing each household with a quality experience.

### Technology adoption and use

We found high levels of initial adoption of the water filter and cookstove through the first year following distribution of the products. Similar rates of reported adoption of both the water filter and cookstove (around 90 %) were seen in the Phase I effort implemented 2 years prior to the large-scale program [10].

Filtered water quantity increased from the pilot study of 1.27 l per person per day to 1.63 l per person per day. The increase may be attributable to increased emphasis in the behavior change program including added messaging about the importance of hydration and specific activities on the household poster which outline how much water should be treated each day in order for the whole family to drink two liters per person day. The high volume of water treated in Rusizi district specifically may be due to increased exposure from recently implemented hygiene and sanitation clubs in only Rusizi district, but further differences between districts are not characterized. However, these differences may be used to customized district level education activities.

Another significant change in the behavior change program was the addition of safe storage messaging. Anecdotal evidence during Phase I suggested that households desired additional storage inside the home and especially while away from the home. In the *Tubeho Neza* program, the majority of households reported storing filtered water with over 80 % storing in a container with a lid, thus emphasizing the importance of the added messaging. Still, about a third of surveyed households reported drinking untreated water while away from the home, mostly while traveling. Given evidence that drinking untreated water, even occasionally, can reduce health benefits of water quality interventions [17], continued emphasis on the importance of safe storage and exclusive consumption of filtered drinking water should be promoted within the program.

**Fig. 6:**
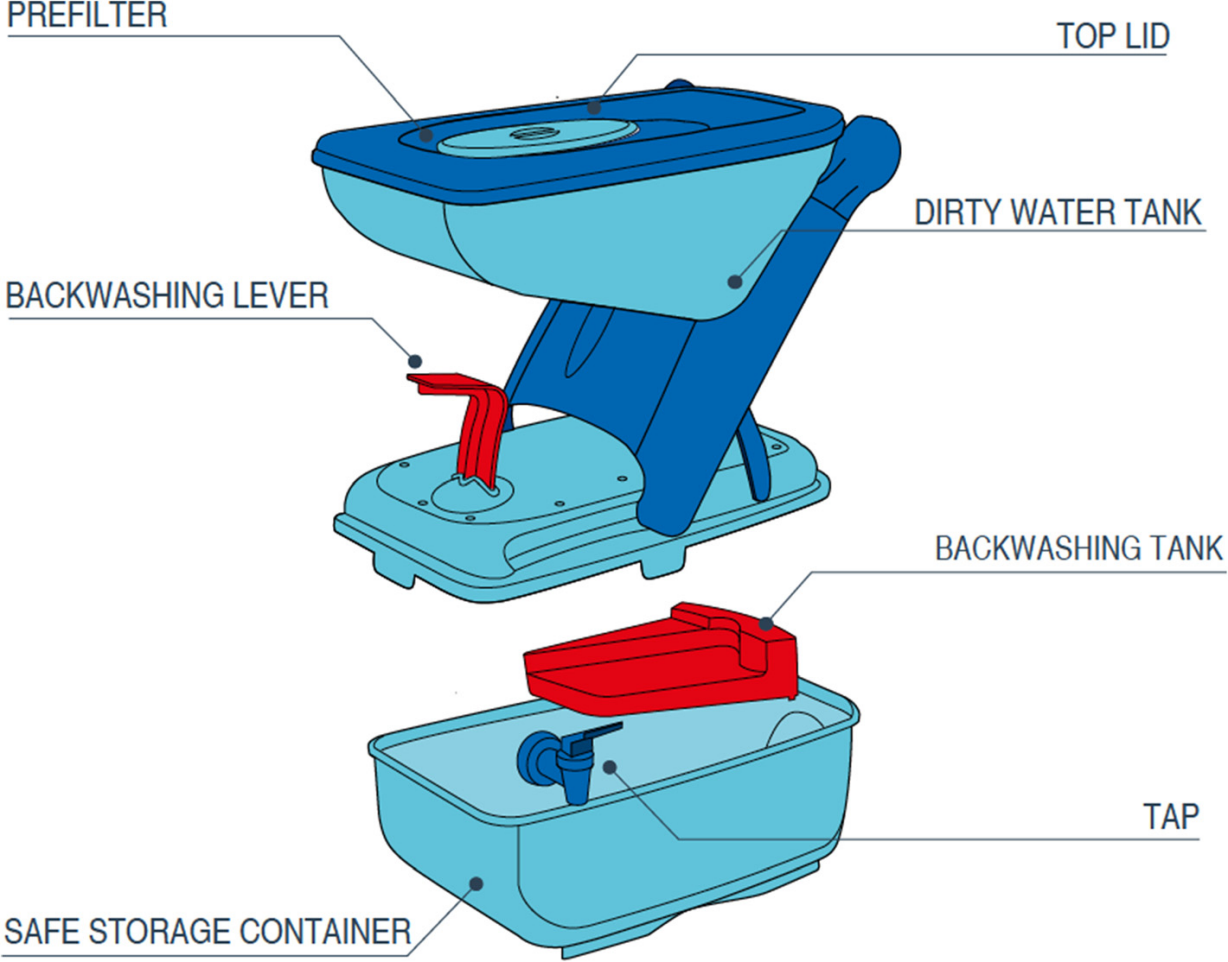
LifeStraw Family 2.0 Diagram. A ultrafiltration membrane cartridge (not pictured) sits above the safe water storage container

While current repairs and replacements of water filters have been less than 2 % of the total households, long term adoption will likely only be realized if filters are continually maintained in a timely manner with an efficient supply chain. Currently repairs are mostly performed by program staff but in order to create a sustainable maintenance structure, local repairs will be needed. The program is currently training CHWs to perform more repairs and solve some maintenance issues. Additionally, one of the more frequent repairs is simply from filters being clogged, likely from these households not backwashing the filter enough. More stress will need to be placed on this maintenance task in future trainings to prevent further clogging issues. Figure 6 shows the components of the water filter, some of which require repair and/or replacement.

While overall reported stove adoption was comparable to the pilot, improvements were made in stove stacking behavior. Reported use of other stoves reduced by over 20 % to about half of households reporting still using other stoves, with percentage of cooking events on the EcoZoom stove in the household increasing by at least 15 %. While these results are promising in moving towards exclusive adoption of improved stoves, they will not be sufficient in meeting the World Health Organization’s guidelines for indoor air pollution [18] which would involve switching to much cleaner fuels and stoves in order to meet recommendations. However, recent evidence suggests that stove interventions may be evaluated based on both the fuel/stove combination and program usage rates, as health gains can be made with lower performing stoves when usage rates are high [19].

One suggested solution to address stove stacking is to provide larger households a second improved stove as many households report desiring a second improved cookstove. However, there was no apparent correlation between household size and stove stacking behavior.

Interestingly number of stove cooking events was highest in Rubavu district, the most urban district in Western Province with the highest rate of charcoal usage. Speculatively, it’s plausible this use is associated with reduced cost of purchasing charcoal fuel.

Another frequently reported behavior change barrier during the pilot was the inability to cook on the EcoZoom stove when fuel was wet. Wood storage messaging to promote drying of wood before households needed fuel for cooking was added and promoted highly through the education and training materials, resulting in the majority households reporting storing wood and over 65 % having dry wood present in their household at the time of the visit. However the primary reported reason for not only using the EcoZoom stove was still a household’s inability to find dry fuel for the EcoZoom.

Rates of outdoor cooking additionally improved from the pilot with 20 % higher frequency of outdoor cooking observed during household visits. A common issue during the pilot was the inability to cook outdoors while it was raining and thus cooking in the doorway as an alternative cooking location was highly emphasized during household visits, where many households reported the doorway as their primary cooking location. The behavior change of cooking outdoors may provide additional important health benefits. The potential for reductions in exposure from cooking outdoors were highlighted in the Phase I RCT study where mean PM_2.5_ concentrations were reduced by 39 % for those cooking indoors on the EcoZoom with further reductions of 73 % when cooking outdoors on the EcoZoom [11].

Free distribution of health products is often debated, centered around claims that free products do not result in adoption rates needed to realize health benefits. This phase 2 program suggests that free distribution is not a determinant of low adoption, consistent with the program design assumptions trialed in the phase 1 program [10]. It is plausible that continued engagement in households, enabled by Ministry of Health support and carbon financed revenue, contributed to high adoption rates. Overall, the *Tubeho Neza* program was able to demonstrate a privately financed, public health intervention can achieve high levels of initial adoption and usage of household level water filtration and improved cookstoves at a large scale.

## Abbreviations

CDM, clean development mechanism; CHW(s), community health worker(s); CI, confidence interval; CO, carbon monoxide; LPPD, liters per person per day; MOH, Rwanda Ministry of Health; PM, particulate matter; RCT, randomized controlled trial; RNEC, Rwanda National Ethics Committee; SRS, simple random sampling; VS, verification survey

## Additional file

Additional file 1: Table S1. Product Tracking Indicators. **Table S2.** Detailed Water Filter Indicators. **Table S3.** Detailed Improved Cookstove Indicators. (DOC 334 kb)
