# Supplementary material for: Process evaluation and assessment of use of a large scale water filter and cookstove program in Rwanda
Source: BMC Public Health. 2016 Jul 16;16:584. doi: 10.1186/s12889-016-3237-0 (PMC4947312; doi:10.1186/s12889-016-3237-0)
Supplement: Additional file 1: Table S1. — Product Tracking Indicators. Table S2. Detailed Water Filter Indicators. Table S3. Detailed Improved Cookstove Indicators. (DOC 334 kb) [file 12889_2016_3237_MOESM1_ESM.doc]

Table S1: Product Tracking Indicators

|  | **During Distribution** | | | **CHW Follow Up Campaign** | | **DelAgua Staff Repairs (up to 9 Months Post Distribution)** | |
| --- | --- | --- | --- | --- | --- | --- | --- |
|  | **n** | **%** | | **n** | **%** | **n** | **%** |
| **Product Repossessions** | 212 | 0.2% | |  |  |  |  |
| Distributed to Wrong Household | 119 | 0.1% | |  |  |  |  |
| Received Multiple Technologies | 59 | 0.1% | |  |  |  |  |
| Sold | 17 | 0.02% | |  |  |  |  |
| Stolen | 4 | 0.004% | |  |  |  |  |
| Other | 13 | 0.01% | |  |  |  |  |
| **EcoZoom Stove** | | | | | | | |
| **Missing Stoves** |  |  | | 1164 | 1.2% |  |  |
| **Location of Missing Stove** |  |  | |  |  |  |  |
| Stolen |  |  | | 335 | 0.3% |  |  |
| Sold |  |  | | 315 | 0.3% |  |  |
| Relative or Neighbors Home |  |  | | 263 | 0.3% |  |  |
| Locked Room |  |  | | 210 | 0.2% |  |  |
| Other |  |  | | 41 | 0.04% |  |  |
| **Stoves Replaced** |  |  | |  |  | 2 | 0.002% |
| Ceramic Cracked |  |  | |  |  | 2 | 0.002% |
| **Repairs Made to Stoves** |  |  | |  |  | 7 | 0.01% |
| Stick Support Replaced |  |  | |  |  | 3 | 0.003% |
| Potskirt Screw Missing |  |  | |  |  | 1 | 0.001% |
| Screws Loose Attaching Stove Together |  |  | |  |  | 3 | 0.003% |
| **LifeStraw Filter 2.0** | | | | | | | |
| **Missing Filters** |  | |  | 930 | 0.9% |  |  |
| **Location of Missing Filters** |  | |  |  |  |  |  |
| Stolen |  | |  | 138 | 0.1% |  |  |
| Sold |  | |  | 261 | 0.3% |  |  |
| Relative or Neighbors Home |  | |  | 208 | 0.2% |  |  |
| Locked Room |  | |  | 254 | 0.3% |  |  |
| Other |  | |  | 56 | 0.1% |  |  |
| **Filters Replaced** |  | |  |  |  | 187 | 0.2% |
| Broken Water Nozzle |  | |  |  |  | 83 | 0.1% |
| Broken Joint Between Tanks |  | |  |  |  | 36 | 0.04% |
| Water Not Passing Through Filter |  | |  |  |  | 24 | 0.02% |
| Safe Water Tank Cracked |  | |  |  |  | 23 | 0.02% |
| Defective/Missing Parts |  | |  |  |  | 12 | 0.01% |
| Leaking |  | |  |  |  | 11 | 0.01% |
| Backwashing Lever Broken |  | |  |  |  | 6 | 0.01% |
| Other |  | |  |  |  | 10 | 0.01% |
| **Repairs Made to Filters** |  | |  | 1460 | 1.5% | 931 | 0.9% |
| Defective/Missing Parts |  | |  | 252 | 0.3% | 678 | 0.7% |
| Backwashing Tube Replaced |  | |  | 0 | 0.0% | 649 | 0.7% |
| Tap |  | |  | 55 | 0.1% | 5 | 0.01% |
| Backwashing Tank |  | |  | 60 | 0.1% | 6 | 0.01% |
| O-ring |  | |  | 99 | 0.1% | 10 | 0.01% |
| Prefilter - Wash and Unclog |  | |  | 38 | 0.04% | 1 | 0.001% |
| Other |  | |  | 0 | 0.0% | 7 | 0.01% |
| Backwash Multiple Times to Unclog Filter |  | |  | 590 | 0.6% | 117 | 0.1% |
| Reassemble Leaking Filter |  | |  | 567 | 0.6% | 67 | 0.1% |
| Joint Between Tanks Reattached |  | |  | 0 | 0.0% | 26 | 0.03% |
| Other |  | |  | 11 | 0.0% | 43 | 0.04% |

Table S2: Detailed Water Filter Indicators

|  | **Verification Round 1 - 6 weeks to 6 months after distribution** | | | **Verification Round 2 - 10 months to 1 year after Distribution** | | |
| --- | --- | --- | --- | --- | --- | --- |
| **Use and Adoption Metrics** | | | | | | |
|  | **n or value** | **%** | ±**95% CI** | **n or value** | **%** | ±**95% CI** |
| **Reported Last Time Filter was Filled** |  |  |  |  |  |  |
| Today | 434 | 44.8% | 3.08% | 74 | 41.8% | 7.07% |
| Yesterday | 414 | 42.8% | 3.07% | 79 | 44.6% | 7.12% |
| Two Days Ago | 74 | 7.6% | 1.65% | 11 | 6.2% | 3.46% |
| More than Two Days Ago | 40 | 4.1% | 1.23% | 13 | 7.3% | 3.74% |
| Don't Know | 6 | 0.6% | 0.49% | 0 | 0.0% | 0.00% |
| **Household Demonstration of Use** |  |  |  |  |  |  |
| Excellent | 487 | 48.9% | 3.10% | 81 | 43.8% | 7.11% |
| Proficient | 0 | 0.0% | 0.00% | 0 | 0.0% | 0.00% |
| Sufficient | 484 | 48.6% | 3.10% | 99 | 53.5% | 7.15% |
| Insufficient | 25 | 2.5% | 0.97% | 5 | 2.7% | 2.32% |
| **Exclusive Use** | | | | | | |
| **Reported Reason for Not Drinking Treated Water** |  |  |  |  |  |  |
| Habit | 12 | 31.6% | 2.88% | 6 | 33.3% | 6.76% |
| Damaged Filter | 8 | 21.1% | 2.53% | 3 | 16.7% | 5.34% |
| No Filtered Water Available at Home | 7 | 18.4% | 2.40% | 2 | 11.1% | 4.50% |
| Working Away from Home | 5 | 13.2% | 2.10% | 3 | 16.7% | 5.34% |
| Don't Know How to Use the Filter | 3 | 7.9% | 1.67% | 0 | 0.0% | 0.00% |
| Other | 3 | 7.9% | 1.67% | 4 | 22.2% | 5.96% |
| **Reported Reason for Not Using LifeStraw** |  |  |  |  |  |  |
| Filter Doesn't Work | 4 | 33.3% | 2.92% | 0 | 0.0% | 0.00% |
| Don't Know How to Use the Filter | 3 | 25.0% | 2.68% | 0 | 0.0% | 0.00% |
| Other | 3 | 25.0% | 2.68% | 1 | 100.0% | 0.00% |
| **Water Use by Members Outside Household** | | | | | | |
| **Reported Giving Filtered Water to People Outside Household** | 699 | 72.2% | 2.78% | 113 | 63.8% | 6.89% |
| **Reported Frequency of Giving Filtered Water to People Outside Household** |  |  |  |  |  |  |
| Usually | 141 | 20.2% | 2.49% | 19 | 16.8% | 5.36% |
| Sometimes | 461 | 66.0% | 2.94% | 54 | 47.8% | 7.16% |
| Rarely | 97 | 13.9% | 2.14% | 40 | 35.4% | 6.85% |
| **Maintenance** | | | | | | |
| **Reported Backwashing Frequency** |  |  |  |  |  |  |
| Everytime Water is Filtered | 924 | 95.5% | 1.29% | 169 | 95.5% | 2.98% |
| Daily | 19 | 2.0% |  | 3 | 1.7% | 1.85% |
| Less than Once per Day | 20 | 2.1% |  | 4 | 2.3% | 2.13% |
| Never | 5 | 0.5% |  | 1 | 0.6% | 1.07% |
| **Water Quantity** | | | | | | |
| **Reported Using Filter for Other Purposes** | 175 | 18.1% | 2.39% | 28 | 15.8% | 5.23% |
| Cleaning the Filter | 68 | 38.9% | 3.02% | 15 | 53.6% | 7.15% |
| Washing Dishes | 54 | 30.9% | 2.86% | 6 | 21.4% | 5.88% |
| Cooking | 35 | 20.0% | 2.48% | 3 | 10.7% | 4.43% |
| Other | 18 | 10.3% | 1.88% | 4 | 14.3% | 5.02% |
| **Reported Water Quantity for Purposes Other than Consumption (lppd)** | 0.479 (SD: 0.389) |  |  | 0.75 (SD: 1.32) |  |  |
| **Total Mean Water Quantity for Purposes Other than Consumption (lppd)** | 0.046 (SD: 0.181) |  |  | 0.052 (SD: 0.386) |  |  |
| **Reported Increase in Consumption of Drinking Water** |  | 140% (SD: 39%) |  |  | 161% (SD: 11%) |  |
| **Safe Storage** | | | | | | |
| **Reported Storage Container Cleaning Frequency** |  |  |  |  |  |  |
| More than Once a Week | 479 | 72.2% | 2.78% | 51 | 44.7% | 7.13% |
| Once a Week | 164 | 24.7% | 2.67% | 58 | 50.9% | 7.17% |
| Less than Once a Week | 18 | 2.7% | 1.01% | 5 | 4.4% | 2.94% |
| Don't Clean the Safe Storage Container | 2 | 0.3% | 0.34% | 0 | 0.0% | 0.00% |
| **Reported Method to Clean Safe Storage Container** |  |  |  |  |  |  |
| With Filtered Water | 409 | 43.3% | 3.07% | 80 | 55.6% | 7.12% |
| Untreated Water | 231 | 24.4% | 2.66% | 32 | 22.2% | 5.96% |
| Soap | 166 | 17.6% | 2.36% | 16 | 11.1% | 4.50% |
| Scrubber/Brush | 45 | 4.8% | 1.32% | 7 | 4.9% | 3.08% |
| Boiled Water | 59 | 6.2% | 1.50% | 8 | 5.6% | 3.28% |
| Other | 35 | 3.7% | 1.17% | 1 | 0.7% | 1.19% |
| **Storage Container have Safe Storage Symbol Present** | 604 | 91.1% | 1.76% | 90 | 78.9% | 5.84% |
| **User Feedback** | | | | | | |
| **Filter Improvements** |  |  |  |  |  |  |
| Nothing | 812 | 73.0% | 2.75% | 140 | 54.1% | 7.14% |
| Increase the Volume | 84 | 7.5% | 1.64% | 39 | 15.1% | 5.13% |
| Add a Stand on the Bottom of the Filter | 66 | 5.9% | 1.46% | 13 | 5.0% | 3.13% |
| Provide Cleaning Accessory | 49 | 4.4% | 1.27% | 18 | 6.9% | 3.64% |
| Faster Flow Rate | 27 | 2.4% | 0.95% | 19 | 7.3% | 3.74% |
| Improve Backwashing Container | 23 | 2.1% | 0.88% | 9 | 3.5% | 2.62% |
| Other | 52 | 4.7% | 1.31% | 21 | 8.1% | 3.91% |
| **Like About Filter** |  |  |  |  |  |  |
| Provides Clean Water | 925 | 44.5% | 3.08% | 175 | 39.9% | 7.02% |
| Like the Taste of Filtered Water | 304 | 14.6% | 2.19% | 53 | 12.1% | 4.67% |
| Provides Safe Water Storage | 212 | 10.2% | 1.88% | 55 | 12.5% | 4.74% |
| Saves Wood from Not Boiling | 211 | 10.2% | 1.87% | 47 | 10.7% | 4.43% |
| Filters Water Quickly | 111 | 5.3% | 1.39% | 27 | 6.2% | 3.44% |
| Looks Nice | 87 | 4.2% | 1.24% | 33 | 7.5% | 3.78% |
| Easy to Use | 84 | 4.0% | 1.22% | 15 | 3.4% | 2.60% |
| Filters All Types of Water | 69 | 3.3% | 1.11% | 29 | 6.6% | 3.56% |
| Improves Health | 36 | 1.7% | 0.81% | 0 | 0.0% | 0.00% |
| Other | 39 | 1.9% | 0.84% | 5 | 1.1% | 1.52% |

**Table S3: Detailed Improved Cookstove Indicators**

|  | **Verification Round 1 - 6 weeks to 6 months after distribution** | | | **Verification Round 2 - 10 months to 1 year after Distribution** | | |
| --- | --- | --- | --- | --- | --- | --- |
|  | **n or value** | **%** | ±**95% CI** | **n or value** | **%** | ±**95% CI** |
| **Use and Adoption Metrics** | | | | | | |
| **Household Demonstration of Use** |  |  |  |  |  |  |
| Excellent | 793 | 79.3% | 2.51% | 145 | 77.5% | 5.98% |
| Proficient | 160 | 16.0% | 2.27% | 37 | 19.8% | 5.71% |
| Sufficient | 29 | 2.9% | 1.04% | 3 | 1.6% | 1.80% |
| Insufficient for Use | 18 | 1.8% | 0.82% | 2 | 1.1% | 1.47% |
| **Reported Use of Pot Skirt Per Week** | 8.24 (SD: 4.52) |  |  | 7.94 (SD: 4.04) |  |  |
| **Reported % Usage of EcoZoom with Pot Skirt** |  | 68.9% | 2.87% |  | 67.1% | 6.73% |
| **Reported Reason for Not Using EcoZoom** |  |  |  |  |  |  |
| Don't Know How to Use | 3 | 25.0% | 2.68% | 0 | 0.0% | 0.00% |
| Doesn't Warm the House | 3 | 25.0% | 2.68% | 0 | 0.0% | 0.00% |
| Difficult to use | 2 | 16.7% | 2.31% | 0 | 0.0% | 0.00% |
| Too Small | 1 | 8.3% | 1.71% | 0 | 0.0% | 0.00% |
| Don't Like Cooking | 1 | 8.3% | 1.71% | 0 | 0.0% | 0.00% |
| Worried About Security of Stove | 1 | 8.3% | 1.71% | 0 | 0.0% | 0.00% |
| Keep it Stored Elsewhere | 1 | 8.3% | 1.71% | 0 | 0.0% | 0.00% |
| Don't Use Wood for Cooking | 0 | 0.0% | 0.00% | 1 | 100.0% | 0.00% |
| **Stove Stacking** | | | | | | |
| **Reported Reason for Not Only Using EcoZoom** |  |  |  |  |  |  |
| Hard to Find Dry Fuel for EcoZoom | 194 | 31.2% | 2.87% | 54 | 36.2% | 6.89% |
| Need Multiple Stoves | 156 | 25.1% | 2.69% | 30 | 20.1% | 5.75% |
| To Warm the House | 92 | 14.8% | 2.20% | 24 | 16.1% | 5.27% |
| Some Food is Difficult to Cook on EcoZoom | 33 | 5.3% | 1.39% | 1 | 0.7% | 1.17% |
| Prefer to Use Charcoal | 23 | 3.7% | 1.17% | 8 | 5.4% | 3.23% |
| Need Light Source | 22 | 3.5% | 1.15% | 1 | 0.7% | 1.17% |
| Pot is Too Big for EcoZoom | 22 | 3.5% | 1.15% | 7 | 4.7% | 3.03% |
| Less Time Tending Other Stove | 18 | 2.9% | 1.04% | 12 | 8.1% | 3.90% |
| Don’t Know How to Use EcoZoom | 12 | 1.9% | 0.85% | 2 | 1.3% | 1.65% |
| Children Can't Cook on EcoZoom | 12 | 1.9% | 0.85% | 3 | 2.0% | 2.01% |
| Fuelwood is Too Big to Fit in EcoZoom | 8 | 1.3% | 0.70% | 0 | 0.0% | 0.00% |
| Other | 29 | 4.7% | 1.31% | 7 | 4.7% | 3.03% |
| **Wood Use Reduction** | | | | | | |
| **Reported % Reduction in Wood Bundles** |  | 57.0% (SD: 21.4%) |  |  | 55.1% (SD: 25.2%) |  |
| **Cooking Location** | | | | | | |
| **Reported Reason for Cooking Indoors** |  |  |  |  |  |  |
| Getting Away from the Rain | 201 | 34.5% | 2.95% | 43 | 30.7% | 6.61% |
| Using Stove Which Can't Be Moved Outdoors | 120 | 20.6% | 2.51% | 15 | 10.7% | 4.43% |
| Warming the Home | 71 | 12.2% | 2.03% | 18 | 12.9% | 4.80% |
| Security | 54 | 9.3% | 1.80% | 16 | 11.4% | 4.56% |
| Habit | 51 | 8.8% | 1.75% | 18 | 12.9% | 4.80% |
| Privacy | 24 | 4.1% | 1.23% | 14 | 10.0% | 4.30% |
| For Light | 23 | 4.0% | 1.21% | 5 | 3.6% | 2.66% |
| Other | 38 | 6.5% | 1.53% | 11 | 7.9% | 3.86% |
| **Cooking Fuel** | | | | | | |
| **Reported Method to Obtain Fuelwood** |  |  |  |  |  |  |
| Collect Wood | 740 | 74.1% | 2.71% | 136 | 73.9% | 6.29% |
| Purchase Wood | 158 | 15.8% | 2.26% | 27 | 14.7% | 5.07% |
| Collect and Purchase Wood | 100 | 10.0% | 1.86% | 21 | 11.4% | 4.56% |
| **Reported Storing Wood** | 930 | 93.0% | 1.58% | 172 | 92.0% | 3.89% |
| **Wood Storage Location** |  |  |  |  |  |  |
| Indoor | 565 | 60.8% | 3.03% | 93 | 54.1% | 7.14% |
| Separate Kitchen | 309 | 33.2% | 2.92% | 70 | 40.7% | 7.04% |
| Under House Awning | 31 | 3.3% | 1.11% | 7 | 4.1% | 2.83% |
| Wood Storage House | 20 | 2.2% | 0.90% | 1 | 0.6% | 1.09% |
| Other | 5 | 0.5% | 0.45% | 1 | 0.6% | 1.09% |
| **Dry Wood Present** | 659 | 65.9% | 2.94% | 150 | 80.2% | 5.71% |
| **User Feedback** | | | | | | |
| **Stove Improvements** |  |  |  |  |  |  |
| Nothing | 743 | 66.8% | 2.92% | 112 | 42.4% | 7.08% |
| Increase Stick Support Size | 131 | 11.8% | 2.00% | 37 | 14.0% | 4.98% |
| Larger Stove Top | 70 | 6.3% | 1.51% | 40 | 15.2% | 5.14% |
| Stove Which Uses Multiple Fuels | 68 | 6.1% | 1.49% | 33 | 12.5% | 4.74% |
| Can be Used Indoors | 39 | 3.5% | 1.14% | 25 | 9.5% | 4.20% |
| Provide Additional Pot Skirt | 23 | 2.1% | 0.88% | 5 | 1.9% | 1.95% |
| Improvements to Pot Skirt | 27 | 2.4% | 0.95% | 2 | 0.8% | 1.24% |
| Add Standing Support | 11 | 1.0% | 0.61% | 0 | 0.0% | 0.00% |
| Other | 39 | 3.5% | 1.14% | 10 | 3.8% | 2.74% |
| **Like About Stove** |  |  |  |  |  |  |
| Cooks Fast | 892 | 32.9% | 2.91% | 163 | 32.7% | 6.72% |
| Reduces Wood | 826 | 30.5% | 2.85% | 154 | 30.9% | 6.62% |
| Produces Less Smoke | 536 | 19.8% | 2.47% | 101 | 20.2% | 5.76% |
| Don't Need to Blow on Fire | 147 | 5.4% | 1.40% | 26 | 5.2% | 3.19% |
| Promotes Cleanliness | 96 | 3.5% | 1.15% | 23 | 4.6% | 3.01% |
| Can be Used by All Members of the Family | 69 | 2.5% | 0.98% | 16 | 3.2% | 2.52% |
| Portable | 60 | 2.2% | 0.91% | 8 | 1.6% | 1.80% |
| Can Use Multiple Sized Pots | 36 | 1.3% | 0.71% | 5 | 1.0% | 1.43% |
| Other | 47 | 1.7% | 0.81% | 3 | 0.6% | 1.11% |
